# Hybrid Pulse High-Frequency Voltage Injection Control Algorithm of Sensorless IPMSM for Vehicles

**DOI:** 10.1155/2022/4248643

**Published:** 2022-09-09

**Authors:** Jingbo Wu, Yongwei Wang, Zhijun Guo

**Affiliations:** ^1^College of Vehicle and Transportation Engineering, Henan University of Science and Technology, Luoyang 471003, China; ^2^Henan Key Laboratory of Automobile Energy Conservation and New Energy, Luoyang 471003, China

## Abstract

A hybrid pulse vibration high-frequency voltage signal injection method is proposed to solve the problems that the conventional sensorless control algorithm of vehicle IPMSM may generate a large estimated rotor position error and opposite directions in identifying the polarity of magnetic poles under zero-speed and high-torque starting and low-speed operation. The magnetic pole polarity is identified by the saturation effect of the flux chain by injecting a high-frequency sinusoidal voltage signal and opposite pulse voltage signal into the axis $\hat{d}$ of the assumed coordinate system simultaneously. Subsequently, the position relationship between the assumed $\hat{d}$ axis and the actual *d* axis is studied in accordance with the amplitude of response current to acquire the rotor position and speed information. The simulation and experimental results suggest that the algorithm is capable of accurately identifying the magnetic pole polarity and estimating the rotor position at zero speed and low speeds, starting the motor smoothly at zero speed, and then operating the motor stably at low speeds.

## 1. Introduction

The interior permanent magnet synchronous motor (IPMSM) is characterized by high power density and high torque-rotation speed. IPMSM has been extensively applied in the driving field of new energy vehicles[1, 2] for its significant advantages in reliability, high torque density, and weak magnetic control [3]. The sensorless IPMSM control system has a wider speed range and faster response speeds than the IPMSM control system with position sensors, and it is capable of accurately acquiring rotor position information under harsh working conditions (e.g., high temperature, high humidity, high pressure, and strong magnetism), thus maximizing the reliability of the electric drive system [4]. The conventional IPMSM sensorless control algorithm usually uses the salient pole effect of the motor body, or the algorithm, to inject high-frequency signals into the motor windings, extract rotor position information from the generated current signals, and then determine the polarity of magnetic poles at zero speed, under high torques, and at low speeds. However, the conventional sensorless control algorithm has great errors in rotor position estimation and magnetic pole polarity identification due to load torque pulsation, so it cannot accurately detect the rotor position. Numerous studies have been conducted to solve the above problems. Literature [5] investigated the injection method of a high-frequency sinusoidal voltage signal of pulse vibration and extracted the rotor position from the amplitude of the high-frequency response current. In Literature [6], the pulse vibration high-frequency carrier voltage signal was injected into the coordinate axis of the *α*-*β* stationary coordinate system of the motor. Moreover, the rotor position information was extracted from the high-frequency current amplitude with the bidirectional phase-locked loop. In literature [7], a fixed high-frequency pulse signal injection method was developed in the rotating coordinate system to reduce high-frequency noise and increase the estimation accuracy of rotor position. In literature [8], a high-frequency voltage signal injection method was developed with two different rotation directions and frequencies, increasing the accuracy of rotor position estimation.

A hybrid pulse high-frequency voltage injection control algorithm is designed in this study. The magnetic pole polarity is identified by the saturation effect of the flux chain by injecting a high-frequency sinusoidal voltage signal and an opposite pulse voltage signal into the axis $\hat{d}$ of the assumed coordinate system simultaneously. Subsequently, the position relationship between the assumed $\hat{d}$ axis and the actual *d* axis is studied in accordance with the amplitude of the response current to obtain the rotor position and speed information and ensure that the motor is capable of starting smoothly at zero speed and operating stably at low speeds. Lastly, a simulation model and a bench test platform are built to verify the effectiveness of the proposed algorithm.

## 2. IPMSM High-Frequency Pumping Mathematical Model

The mathematical model of IPMSM's high-frequency excitation should be built first to investigate the hybrid pulse high-frequency voltage signal injection method [9–12]. The voltage equation of IPMSM in the d-q rotating coordinate system is written as

(1) $$\begin{array}{c} \left\{\begin{array}{c} u_{d}=Ri_{d}+\frac{d}{dt}\psi_{d}-\omega_{e}\psi_{q},\\ u_{q}=Ri_{q}+\frac{d}{dt}\psi_{q}+\omega_{e}\psi_{d}, \end{array}\right. \end{array}$$

where *u*_*d*_ and *u*_*q*_ denote the components of the stator voltage on the axis *d* and the axis *q*, respectively; *i*_*d*_ and *i*_*q*_represent the components of the stator current on the axis *d* and the axis *q*, respectively; *ψ*_*d*_ and *ψ*_*q*_ express the components of the stator flux on the axis *d* and the axis *q*, respectively; *R* denotes the stator resistance; *ω*_*e*_ is the electrical angular velocity.

Since the axis *d* is consistent with the central axis of the rotor magnetic pole, the stator flux equation is expressed as

(2) $$\begin{array}{c} \left\{\begin{array}{c} \psi_{d}=L_{d}i_{d}+\psi_{f},\\ \psi_{q}=L_{q}i_{q}, \end{array}\right. \end{array}$$

where *L*_*d*_ and *L*_*q*_ denote the components of the stator inductance on the axis *d* and the axis *q*, respectively.

Equation (1) is transformed into an *α-β* stationary coordinate system, and its voltage equation is expressed as

(3) $$\begin{array}{c} \left[\begin{array}{c} u_{\alpha }\\ u_{\beta } \end{array}\right]=R\left[\begin{array}{c} i_{\alpha }\\ i_{\beta } \end{array}\right]+\frac{d}{dt}\left[\begin{array}{c} \psi_{\alpha }\\ \psi_{\beta } \end{array}\right]\text{,} \end{array}$$

where *u*_*α*_, *u*_*β*_, *i*_*α*_, and *i*_*β*_ represent the stator voltage and current in the *α-β* two-phase static coordinate system, respectively; *ψ*_*α*_ and *ψ*_*β*_ denote the flux linkage in the *α-β* two-phase stationary coordinate system, respectively.

The stator flux equation is written as

(4) $$\begin{array}{c} \left[\begin{array}{c} \psi_{\alpha }\\ \psi_{\beta } \end{array}\right]=\left[\begin{array}{cc} L+\Delta L\;\cos\;2\theta_{e} & -\Delta L\;\sin\;2\theta_{e}\\ -\Delta L\;\sin\;2\theta_{e} & L-\Delta L\;\cos\;2\theta_{e} \end{array}\right]\left[\begin{array}{c} i_{\alpha }\\ i_{\beta } \end{array}\right]+\psi_{f}\left[\begin{array}{c} \cos\theta_{e}\\ \sin\theta_{e} \end{array}\right]\text{,} \end{array}$$

where *L* and Δ*L* represent the average inductance and half-difference inductance, respectively; *ψ*_*f*_ denotes the permanent magnet flux; *θ*_*e*_ is the electrical angle of the rotor position. *L* and Δ*L* are expressed as follows:

(5) $$\begin{array}{c} \left\{\begin{array}{c} L=\frac{L_{d}+L_{q}}{2},\\ \Delta L=\frac{L_{d}-L_{q}}{2}. \end{array}\right. \end{array}$$

The inductance in the *α-β* two-phase stationary coordinate system contains rotor position information *θ*_*e*_ in accordance with equation (4).

## 3. Hybrid Pulse High-Frequency Voltage Signal Injection Method

### 3.1. Estimate Rotor Position with Pulse High-Frequency Voltage Injection Method

**Figure 1:**
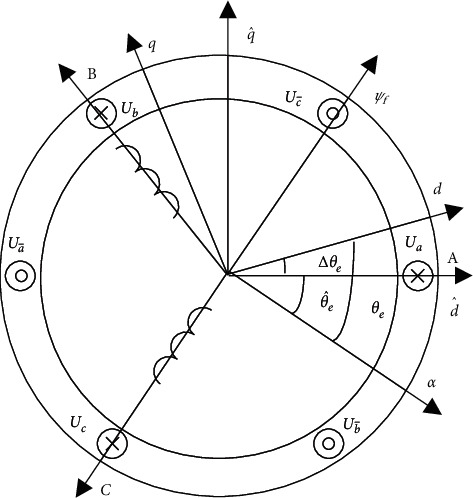
Positional relationship between each coordinate system.

In this study, the pulse high-frequency voltage signal is injected into the straight axis in the $\hat{d}$-$\hat{q}$ hypothetic-synchronous rotation coordinate system to produce an inductance saturation salient pole effect and a high-frequency response current. Afterward, the rotor position information contained in the high-frequency response current is analyzed. It is imperative to assume the $\hat{d}$-$\hat{q}$ hypothetic-synchronous rotation coordinate system to estimate rotor position and speed by the pulse high-frequency voltage signal injection method. The positional relationship between d-q and$\hat{d}-\hat{q}$ coordinate systems is constructed in accordance with the relationship among coordinate systems [13–17], as presented in Figure 1.

In Figure 1, the position error angle of the actual *d-q* synchronous rotation coordinate system and the hypothesized $\hat{d}$-$\hat{q}$ synchronous rotation coordinate system is Δ*θ*_*e*_, the actual rotor position angle is *θ*_*e*_, and the estimated rotor position angle is ${\hat{\theta }}_{e}$, and the relationship between the two is as follows:

(6) $$\begin{array}{c} \Delta \theta_{e}=\theta_{e}-{\hat{\theta }}_{e}\text{.} \end{array}$$

Based on the rotor position error between the assumed rotating coordinate system and the actual rotating coordinate system in (6), the voltage equation in the rotating coordinate system under high-frequency signal excitation is written as

(7) $$\begin{array}{c} \left\{\begin{array}{c} u_{din}\approx L_{\hat{d}}\frac{di_{din}}{dt},\\ u_{qin}\approx L_{\hat{q}}\frac{di_{qin}}{dt}. \end{array}\right. \end{array}$$

It is assumed that the stator inductance in the $\hat{d}$-$\hat{q}$ rotating coordinate system can be expressed as $L_{\hat{d}}、L_{\hat{q}}$, the *L*_*αβ*_ equation in the stationary coordinate system can be derived by (4), and the expression is presented as follows:

(8) $$\begin{array}{c} L_{\alpha \beta }=\left[\begin{array}{cc} L+\Delta L\;\cos\;2\theta_{e} & -\Delta L\;\sin\;2\theta_{e}\\ -\Delta L\;\sin\;2\theta_{e} & L-\Delta L\;\cos\;2\theta_{e} \end{array}\right]\text{.} \end{array}$$

According to (7) and (8), in the assumed synchronous$\hat{d}$-$\hat{q}$ rotation coordinate system, the high-frequency response current equation generated by injecting the pulse high-frequency voltage signal as follows:

(9) $$\begin{array}{c} \left[\begin{array}{c} \frac{d{\hat{i}}_{din}}{dt}\\ \frac{d{\hat{i}}_{qin}}{dt} \end{array}\right]=\left[\begin{array}{cc} \cos\;\Delta \theta_{e} & -\sin\;\Delta \theta_{e}\\ \sin\;\Delta \theta_{e} & \cos\;\Delta \theta_{e} \end{array}\right]\left[\begin{array}{cc} \frac{1}{L_{\hat{d}}} & 0\\ 0 & \frac{1}{L_{\hat{q}}} \end{array}\right]\left[\begin{array}{cc} \cos\;\Delta \theta_{e} & \sin\;\Delta \theta_{e}\\ -\sin\;\Delta \theta_{e} & \cos\;\Delta \theta_{e} \end{array}\right]\left[\begin{array}{c} {\hat{u}}_{di\;n}\\ {\hat{u}}_{qin} \end{array}\right]\text{,} \end{array}$$

where ${\hat{u}}_{di\;n}$, ${\hat{u}}_{qin}$, ${\hat{i}}_{di\;n}$, and ${\hat{i}}_{qin}$ are the high-frequency voltage component and high-frequency current component of the axis $\hat{d}$ and the axis $\hat{q}$ in the $\hat{d}$-$\hat{q}$ coordinate system.

Using the average inductance and semidifference inductance in the hypothesized $\hat{d}$-$\hat{q}$ rotation coordinate system, (9) can be rewritten as

(10) $$\begin{array}{c} \left[\begin{array}{c} \frac{d{\hat{i}}_{din}}{dt}\\ \frac{d{\hat{i}}_{qin}}{dt} \end{array}\right]=\frac{1}{{\hat{L}}^{2}-\Delta {\hat{L}}^{2}}\left[\begin{array}{cc} \hat{L}+\Delta \hat{L}\cos\;2\Delta \theta_{e} & \Delta \hat{L}\sin\;2\Delta \theta_{e}\\ \Delta \hat{L}\sin\;2\Delta \theta_{e} & \hat{L}-\Delta \hat{L}\cos\;\Delta \theta_{e} \end{array}\right]\left[\begin{array}{c} {\hat{u}}_{din}\\ {\hat{u}}_{qin} \end{array}\right]\text{,} \end{array}$$

where $\hat{L}$ and $\Delta \hat{L}$ represent the average inductance and semidifference inductance in the assumed synchronous rotating coordinate system, respectively, which are expressed as follows:

(11) $$\begin{array}{c} \left\{\begin{array}{c} \hat{L}=\frac{L_{\hat{d}}+L_{\hat{q}}}{2},\\ \Delta \hat{L}=\frac{L_{\hat{d}}-L_{\hat{q}}}{2}. \end{array}\right. \end{array}$$

A high-frequency sinusoidal voltage signal ${\hat{u}}_{di\;n}$ is injected into the axis *d* in the synchronous rotating coordinate system based on the above equation, and no injection signal is injected into the intersecting axis at this time, namely,${\hat{u}}_{di\;n}$. Thus, when Δ*θ*_*e*_=0, the response current corresponding to the intersecting axis is ${\hat{i}}_{qin}=0$, and IPMSM does not generate torque pulsation. The high-frequency sinusoidal voltage signal injection method is selected to estimate the rotor position, and the high-frequency injection signal equation is expressed as

(12) $$\begin{array}{c} \left\{\begin{array}{c} {\hat{u}}_{din}=u_{in}\cos\;\left(\omega_{in}t\right),\\ {\hat{u}}_{qin}=0, \end{array}\right. \end{array}$$

where *u*_*in*_ denotes the peak value of the injected high-frequency voltage; *ω*_*in*_ is the angular frequency of the injected high-frequency sinusoidal signal.

The following equation can be obtained from the generated high-frequency response current (10):

(13) $$\begin{array}{c} \left[\begin{array}{c} {\hat{i}}_{din}\\ {\hat{i}}_{qin} \end{array}\right]=\frac{u_{in}\sin\;\left(\omega_{in}t\right)}{\omega_{in}\left({\hat{L}}^{2}-\Delta {\hat{L}}^{2}\right)}\left[\begin{array}{c} \hat{L}+\Delta \hat{L}\cos\;2\Delta \theta_{e}\\ \Delta \hat{L}\sin\;2\Delta \theta_{e} \end{array}\right]\text{.} \end{array}$$

During the injection of the high-frequency signal, $R_{s}\ll \omega_{in}L_{\hat{d}}$, so the impact generated by *R*_*s*_ can be ignored, and the equivalent high-frequency impedance generated can be simplified. The component equation of the high-frequency current generated on the intersecting axis is written as

(14) $$\begin{array}{c} {\hat{i}}_{qin}\approx \frac{u_{in}\sin\;\left(\omega_{in}t\right)\Delta \hat{L}\sin\;2\Delta \theta_{e}}{\omega_{in}\left({\hat{L}}^{2}-\Delta {\hat{L}}^{2}\right)}\text{.} \end{array}$$

Equation (14) suggests that the high-frequency current components of the axis $\hat{d}$ and the axis $\hat{q}$ all contain rotor position information. When the rotor position error Δ*θ*_*e*_ tends to approach 0, the high-frequency current component ${\hat{i}}_{qin}$ on the axis $\hat{q}$ also tends to approach 0. Thus, the rotor position and speed can be obtained by adjusting the high-frequency current on the axis $\hat{q}$ based on (4) using the rotor position tracking observer.

### 3.2. Polarity Identification by Pulse Voltage Injection Method

In this study, the polarity is identified by injecting pulse voltage into the hypothesized $\hat{d}$ axis. IPMSM transforms voltage and current components in the stationary coordinate system in accordance with Euler's theorem to yield the resultant vector equation [18–25], which is expressed as

(15) $$\begin{array}{c} U_{\alpha \beta }=RI_{\alpha \beta }+L\frac{d}{dt}I_{\alpha \beta }+\Delta L\frac{d}{dt}{I_{\alpha \beta }^{*}}^{ej2\theta_{e}}\text{,} \end{array}$$

where **U**_*αβ*_ denotes voltage vector; **I**_*αβ*_ is the current vector; **I**_*αβ*_^*∗*^ represents the conjugate vector of **I**_*αβ*_.

IPMSM is injected with a high-frequency signal, **U**_*αβ*_ ≫ *R ***I**_*αβ*_, so the voltage generated by the stator resistance can be ignored. (15) is approximately equivalent to

(16) $$\begin{array}{c} U_{\alpha \beta }=L\frac{d}{dt}I_{\alpha \beta }+\Delta L\frac{d}{dt}{I_{\alpha \beta }^{*}}^{ej2\theta_{e}}\text{.} \end{array}$$

Next, it is transformed into

(17) $$\begin{array}{c} \frac{d}{dt}I_{\alpha \beta }=\frac{LU_{\alpha \beta }+\Delta L{U_{\alpha \beta }^{*}}^{ej2\theta_{e}}}{L^{2}-\Delta L^{2}}\text{,} \end{array}$$

where **U**_*αβ*_^*∗*^ denotes the conjugate vector of **U**_*αβ*_. In a pulse injection cycle Δ*t*, the current change is expressed as

(18) $$\begin{array}{c} alpha-beta\Delta I_{\alpha \beta }=\frac{L+\Delta Le^{j2\Delta \theta_{e}}}{L^{2}-\Delta L^{2}}\Delta tU_{\alpha \beta }\text{.} \end{array}$$

Converting equation (18) to the hypothesized synchronous $\hat{d}$-$\hat{q}$ rotation coordinate system, which is expressed as

(19) $$\begin{array}{c} \Delta I_{\hat{d}\hat{q}}=\frac{L+\Delta Le^{j2\Delta \theta_{e}}}{L^{2}-\Delta L^{2}}\Delta tU_{\hat{d}\hat{q}}\text{.} \end{array}$$

Based on the salient pole characteristics of IPMSM, the pulse high-frequency sinusoidal voltage signal is injected to acquire current information and obtain the rotor initial angle ${\overset{⌢}{\theta }}_{r}$. The polarity of the rotor magnetic pole can be identified based on the principle of rotor polarity identification. Subsequently, in the hypothesized $\hat{d}$-$\hat{q}$ synchronous rotating coordinate system, an equivalent pulse voltage (equal amplitude, 180°difference in vector angle) is applied to the axis $\hat{d}$ in the opposite direction of the positive and negative direction. The injection hypothesized $\hat{d}$ axis is in the same direction as the actual *d* axis if the magneto-motive force generated by the current deepens the magnetic circuit. If the saturation degree of the magnetic circuit decreases, the injection direction is opposite to the *d* axis. Accordingly, (19) suggests that the rotor magnetic pole polarity can be accurately identified by comparing the amplitude of the pulse current response and the high-frequency sinusoidal current response.

### 3.3. Observation of Rotor Initial Position of One-Phase-Locked Loop

The high-frequency carrier current component contains information (e.g., magnetic pole polarity, rotor position, and speed), which is difficult to be extracted directly from the high-frequency carrier current component. Therefore, the high-frequency current component on the axis $\hat{q}$ should be modulated first. Subsequently, the high-frequency clutter signal is filtered out with a low-pass filter. Lastly, the rotor position is studied by inputting to the rotor position tracking observer.

**Figure 2:**
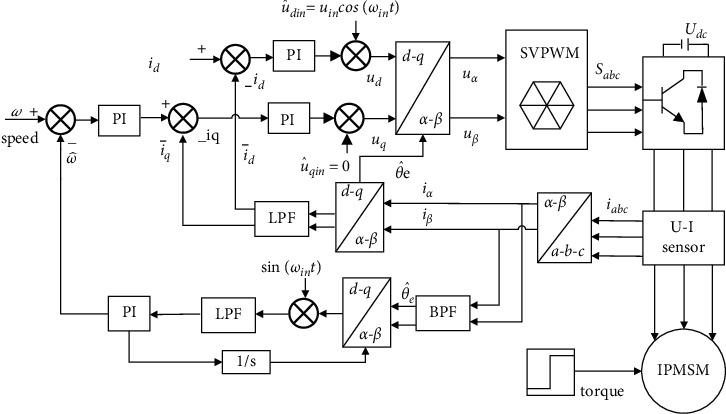
Phase-locked loop-block diagram of the pulse vibration high-frequency voltage signal injection method.

The position error signal is adopted to improve the filter function of the loop filter through the PI regulator, and the accurate estimated speed value is obtained in real-time. Next, the integral regulator is adopted to achieve the function of VCO and adjust the estimated rotor position by feedback.  Figure 2 illustrates the control principle based on the phase-locked loop-pulse high-frequency voltage signal injection method.

To obtain accurate rotor position and speed, the PLL control system is reconstructed with a PI regulator and second-order low-pass filter. The expected bandwidth of the system filter is *σ*, and the transfer function of the PI regulator and the second-order low-pass filter is

(20) $$\begin{array}{c} \left\{\begin{array}{c} F\left(s\right)=\frac{\sigma^{2}}{s^{2}+3s\sigma +\sigma^{2}},\\ G\left(s\right)=k_{p}+\frac{k_{s}}{s}, \end{array}\right. \end{array}$$

where *k*_*p*_ and *k*_*s*_ are the proportional gain coefficient and integral gain coefficient of the PI regulator, respectively.

## 4. Simulation and Bench Test

### 4.1. Simulation Analysis

To verify the accuracy and reliability of the sensorless control algorithm designed for IPMSM under zero-speed starting and low-speed conditions, a simulation model of the IPMSM sensorless vector control system is established by using the MATLAB/Simulink software, and the simulation analysis is carried out under variable load and speed conditions.  Figure 2 illustrates the control principle, and Table 1 lists the parameters of the driving motor and the control system. The simulation conditions consist of the ode45 algorithm with a fixed step size, a sampling time of 10^−6^ s, and a simulation time of 0.5 s. Moreover, the amplitude of the pulse high-frequency voltage injection signal is set to *V*_*in*_=25 V and the frequency of the injection high-frequency voltage signal is *f*_*in*_=1000 Hz. At this point, the pulse high-frequency voltage signal equation is expressed as*u*_*in*_=*V*_*in*_cos (2*πf*_*in*_*t*).

The high-pass and low-pass filters in this algorithm adopt a second-order filter, thus more effectively suppressing the high-frequency or low-frequency components and interference noise contained in the high-frequency excitation signal and increasing the accuracy of the rotor position and speed estimation.

#### 4.1.1. Analysis of No-Load Starting and Constant Speed Operation

**Figure 3:**
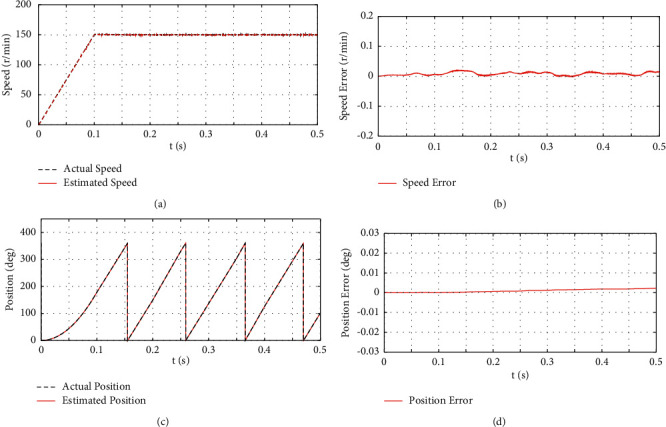
The simulation results of starting without load and constant speed. (a) The waveform of actual and estimated rotational speed values. (b) The waveform of speed estimation error. (c) The waveform of actual and estimated rotor positions. (d) The waveform of rotor position error.

The initial torque of the no-load starting motor is set to 0 N·m. When *t* = 0.2 s, the motor suddenly loads a torque of 5 N·m, and when *t* = 0.4 s, the load is unloaded. The motor's initial speed is 0 r/min, and it is uniformly accelerated to 150 r/min within *t*∈(0, 0.1)*s*, and it runs at constant speed until the end of the simulation.  Figure 3 depicts the estimated rotor position, rotational speed, rotor position error, and rotational speed error of the proposed algorithm under load disturbance.

Figure 3 presents the simulation results of the sensorless control algorithm for IPMSM hybrid high-frequency signal injection under the no-load start-constant speed condition. As depicted in Figures 3(a) and 3(b), this algorithm is capable of accurately estimating the speed, and the estimated speed can be well consistent with the actual speed. The maximum error of the estimated speed is nearly 0.02 r/min, and the estimation effect is good. Moreover, Figures 3(c) and 3(d) suggest that the proposed algorithm is capable of accurately estimating the rotor position, which can follow the actual rotor position, with the maximum error of the estimated rotor position angle reaching nearly 0.002 deg. As revealed by the above results, the designed algorithm can provide a more accurate rotor position for the sensorless control system, and IPMSM can start smoothly at zero speed and operate smoothly at low speeds.

#### 4.1.2. Variable Speed and Load Operation Analysis

**Figure 4:**
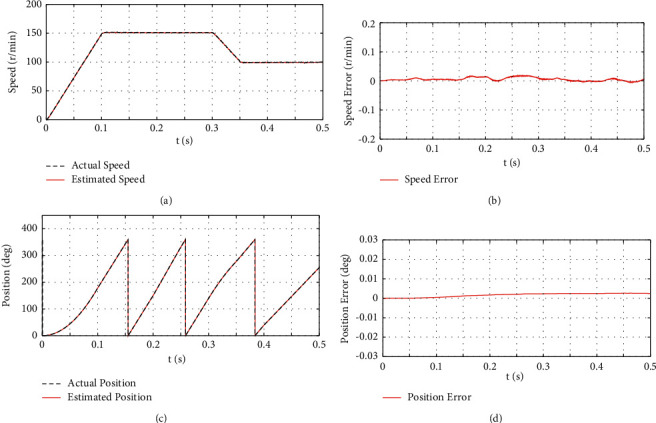
The simulation results of variable speed and variable load. (a) The waveform of actual and estimated rotational speed values. (b) The waveform of speed estimation error. (c) The waveform of actual and estimated rotor positions. (d) The waveform of rotor position error.

The motor's initial torque is set to 0 N·m, and a load torque of 5 N·m is loaded in *t*∈(0.1, 0.3) *s*. When *t* = 0.3 s, the load is unloaded, and a load torque of 5 N·m is loaded in *t*∈(0.35, 0.5) *s*. The motor has an initial speed of 0 r/min and is uniformly accelerated to 150 r/min in *t*∈(0, 0.1) *s*. Subsequently, the motor runs at a constant speed in *t*∈(0.1, 0.3) *s*. The motor is uniformly decelerated to 100 r/min in *t*∈(0.3, 0.35) *s* and then runs at a uniform speed until the end of the simulation.  Figure 4 presents the estimated and actual rotor position, speed, rotor position error, and speed error of the proposed algorithm under speed disturbance.

Figure 4 depicts the simulation results of the IPMSM hybrid high-frequency signal injection sensorless control algorithm at varying speeds and under changing loads. As depicted in Figures 4(a) and 4(b), the algorithm can still accurately estimate the speed under varying speeds and changing loads. When the speed changes and the load torque is loaded, the fluctuation of the speed error increases, whereas the maximum error is 0.02 r/min. As depicted in Figures 4(c) and 4(d), the proposed algorithm is capable of accurately estimating the rotor position under varying speeds and changing loads. With varying speeds and the loading of the load torque, the rotor position angle error increases when the torque varies, and the maximum error of the rotor position angle is estimated to be nearly 0.0025 deg. As revealed by the results, the proposed algorithm is capable of starting IPMSM smoothly at zero speed and operating smoothly under acceleration, deceleration, and load mutation while only small errors are generated in the estimated speed and rotor position.

### 4.2. Bench Test Analysis

**Figure 5:**
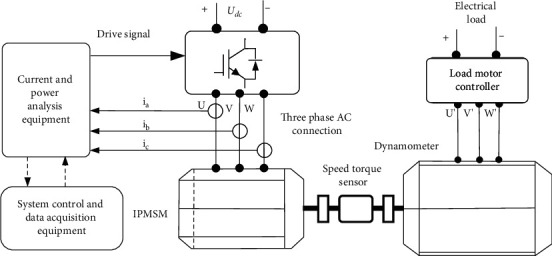
The principal block diagram of the IPMSM bench test system.

The magnetic pole polarity judgement and the low-speed sensorless control algorithm developed in this study, the principal block diagram of the bench test system is illustrated in Figure 5 to further verify the accuracy and reliability of the zero-velocity initial position estimation.

**Figure 6:**
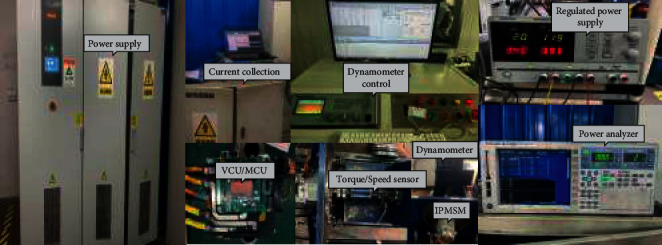
The test platform of the IPMSM drive control system.

Table 1 lists the IPMSM parameters required for the bench test. A sensorless vector drive control platform is built in accordance with the schematic diagram of the IPMSM bench test system (Figure 6). The test equipment in Figure 6 comprises a DC power cabinet, vehicle controller, motor controller, power analyzer, dynamometer, oscilloscope, torque and speed sensors, upper computer software, test bench, etc. A TMS320F28335 32-bit floating-point DSP processor is adopted in the motor control chip, and the vehicle controller chip adopts an NXP MPC5744P processor with PA architecture.

**Figure 7:**
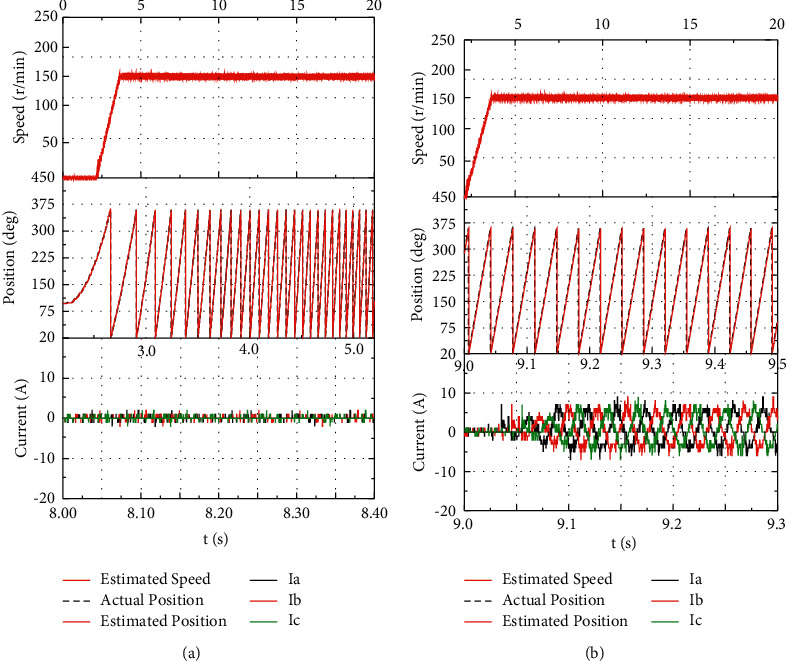
The test results of IPMSM at zero speed and low speeds. (a) No-load operation. (b) Loaded operation.

In the bench test, the frequency and amplitude of the high-frequency voltage injected into the IPMSM stator windings are set to 1 k·Hz and 25 V, respectively. To verify the accuracy of the algorithm for speed and rotor position estimation, the motor speed is increased from 0 to 150 r/min, and a load of 5 N·m is loaded after the steady operation of the motor at a constant speed.  Figure 7 illustrates the waveform of speed, rotor position, and three-phase current (part).

The bench test results suggest that when the IPMSM starts, the rotor position is a random position angle, and the proposed algorithm can accurately estimate the initial rotor position. As revealed by the current waveform, although IPMSM has a slight jitter when starting, the motor can still be ensured to start smoothly. When the motor operates smoothly, a load torque of 5 N·m suddenly increases, the speed is estimated to be stable at nearly 150 r/min, and the motor has a fast current response. The bench test results suggest that the proposed algorithm is capable of effectively estimating the rotor position and speed at zero speed and low speeds and that the IPMSM sensorless control system can operate smoothly at low speeds.

## 5. Conclusion

Under the working condition that vehicles start at zero speed, under high torque, and at low speeds, a hybrid pulse vibration high-frequency voltage signal injection method is proposed to solve the problems that the conventional sensorless control algorithm of vehicle IPMSM may produce a large estimated rotor position error and opposite directions of identifying the polarity of magnetic poles under zero-speed and high-torque starting and low-speed operation. The simulation results indicate that the maximum errors of the rotor position angle are 0.0025°deg and 0.002°deg, respectively, and the estimated maximum errors of the rotor speed are 0.02 r/min. The result of the bench test further verifies that the proposed algorithm is capable of accurately estimating rotor position and identifying magnetic pole polarity at zero speed and low speeds and that IPMSM can start smoothly at zero speed and operate stably at low speeds.

## Figures and Tables

**Table 1:** Parameters of IPMSM and control system.

Name	Parameter	Numerical number	Unit	Name	Parameter	Numerical number	Unit
Stator resistance	*R *_s_	0.24	Ohm	Rated torque	*T *_N_	25	N·m
Stator flux linkage	Ψ_*f*_	0.13	Wb	Pole-pairs	*N *_p_	4	P
*d-*axis inductance	*L *_d_	2.37	mH	Rated power	*P *_N_	7.9	kW
*q-axis inductance*	*L *_q_	5.94	mH	Rated speed	n_*ref*_	3000	r/min
DC power supply	*U *_dc_	300	V	Rotational inertia	*J*	0.008	kg.m^2^
Rated voltage	*U *_N_	310	V	Frequency	*f*	10000	Hz
Rated current	*I *_N_	17.8	A				

## Data Availability

The dataset can be accessed upon request.
